# Assessment of hygiene practices and microbial safety of milk supplied by smallholder farmers to processors in selected counties in Kenya

**DOI:** 10.1007/s11250-022-03214-7

**Published:** 2022-06-29

**Authors:** Miriam W. Mogotu, George O. Abong, John Mburu, Oghaiki Asaah Ndambi

**Affiliations:** 1 Department of Food Science Nutrition and Technology, University of Nairobi, P.O. Box 29053 – 00625, Kangemi, Kenya; 2 Department of Agricultural Economics, University of Nairobi, P.O. Box 29053 - 00625, Kangemi, Kenya; 3 Animal Science Group, Wageningen University & Research, P.O. Box 338, 6700 AB Wageningen, Netherlands

**Keywords:** Collection channels, *E. coli*, Hygiene, Kenya, Milk quality, TVC, *Staphylococcus*

## Abstract

Smallholder farmers dominate the Kenyan dairy sector producing 95% of the total milk. However, several concerns have been raised on the quality and safety of the milk they produce. This study assessed the hygienic practices and microbial safety of milk supplied by smallholder farmers to processors in Bomet, Nyeri, and Nakuru counties in Kenya. Interviews and direct observations were carried out to assess hygiene and handling practices by farmers and a total of 92 milk samples were collected along four collection channels: direct suppliers, traders, cooperatives with coolers, and cooperatives without coolers. Microbial analysis was done following standard procedures and data analysed using GenStat and SPSS. This study revealed that farmers did not employ good hygienic practices in their routine dairy management. They used plastic containers for milking and milk storage (34.2%); they did not clean sheds (47.9%) and did not set aside cows that suffered from mastitis factors (83.6%), resulting in poor microbial quality of raw milk along the collection channels. The highest mean total viable counts (8.72 log_10_ cfu/ml) were recorded in Nakuru while Nyeri had the highest mean *E. coli* counts (4.97 log_10_ cfu/ml) and Bomet recorded the highest mean counts of 5.13 and 5.78 log_10_ cfu/ml for *Staphylococcus aureus* and *Listeria monocytogenes* respectively. Based on all above-mentioned parameters, the microbial load in most samples from all three counties exceeded the set Kenyan standards. Farmer training, improving road infrastructure, use of instant coolers at cooperatives, and quality-based payment systems are recommended as measures to curb microbial growth.

## Introduction

Kenya’s dairy sector has a significant socio-economic role in the national economy. It is a source of livelihood and nutrition for many, generating about 4% of the national GDP and has been ranked among the largest in sub-Saharan Africa. Small-scale farmers dominate the dairy industry at production level where they produce over 95% of the national milk (KDB 2020). Dairy production in Kenya is mainly practiced in the highlands and is mostly intensive or semi-intensive farming (Bonilla et al. 2018). Dairy herds in Kenya comprise of an estimated 4.5 million head of pure-bred Fresian Holstein, Ayshire, Guernsey, Jersey, and other crosses which produce over 5.25 billion litres of milk per year (KDB 2020).

Milk produced by small-scale dairy farmers is consumed both in urban and rural areas and is a necessity for most Kenyans. Milk has a relatively short shelf life thus requires quick and efficient marketing to assure its safety and quality There has been great emphasis on the organization of small-scale milk producers into groups such as self-help groups, cooperatives, and companies to enhance efficiency in the marketing of raw milk, with dairy cooperatives dominating the marketing of milk (KDB 2020). Milk processing capacity in Kenya is also on a steady growth owing to the growing demand of milk and dairy products with new milk processors coming up in different counties and sourcing milk from farmers within the community (KNBS 2020).

Despite the increased in demand, processing, and marketing of milk, there still remains a challenge of noncompliance with national, regional, and international quality and safety standards (Bebe et al. 2018). This is mainly due to the lack of efficient monitoring and proper enforcement structures in the country.

Milk marketed in the formal and informal sectors in Kenya often does not meet the set microbial standards, posing a health hazard (Knight-jones et al. 2016). Milk and dairy products are rich in nutrients making them a good environment for the growth of both spoilage and pathogenic microorganisms (Alonso and Grace 2018).

To reduce milk spoilage, more dairy cooperatives have been established where farmers bulk and cool their milk before it is marketed or transported to processors (Odero-Waitituh 2017). There has also been an increase in the number of middlemen or traders who bulk milk from several farmers and transport it to the cooperatives or processors. While some have helped in ensuring the efficiency of milk transportation, most have brought more complications in the traceability of milk (Bonilla et al. 2018).

There is a need to assess the hygiene knowledge and handling practices of milk by farmers, considering that milk contamination usually begins at the production level and given that the microbial safety of raw milk in Kenya from small-scale farmers has been a grave concern for decades (Knight-jones et al. 2016; Alonso et al. 2018; Brown et al. 2018). Understanding the various socio-demographic characteristics of the respondents will provide additional knowledge on how they influence hygiene knowledge and handling practices of milk by farmers. The presence of middlemen or traders further complicates the traceability of milk and brings a risk of cross-contamination and microbial overload due to poor milk handling by transporters, adulteration of milk, and in some cases long transportation time without refrigeration (Vara Martínez et al. 2017). There is also limited data on the microbial quality of milk along collection channels despite the need for monitoring from production to consumption (Ndungu et al. 2016a).

## Materials and methods

### Study site

**Fig. 1:**
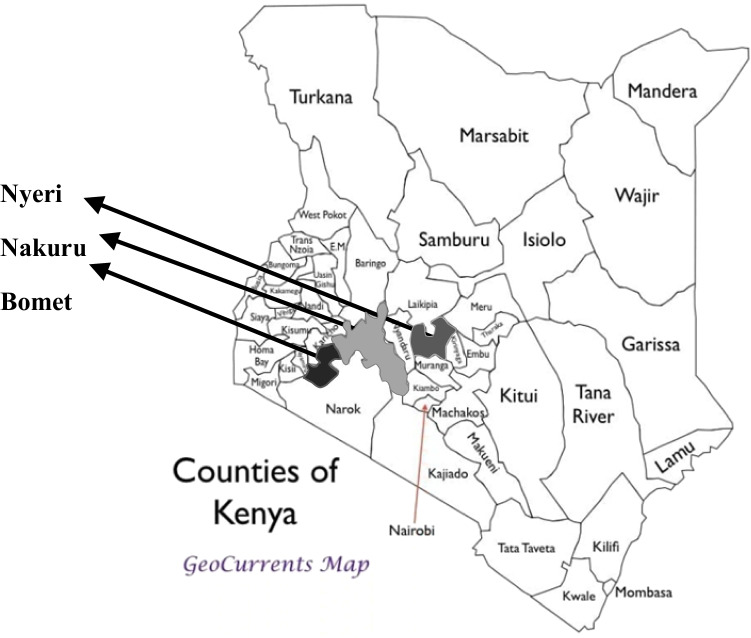
Map of Kenya showing Bomet, Nakuru, and Nyeri counties. Source: GeoCurrents Map, 2020

The study was carried out in Bomet, Nakuru, and Nyeri counties in Kenya (Fig. 1). Agriculture is the main economic activity in the three counties with dairy farming dominating (KDB 2020). In Bomet, most farmers practice semi-intensive dairy farming where cows are allowed to graze in the fields during the day and housed in stalls at night (CGOB 2019) due to larger size of land in the area. In Nakuru and Nyeri, farmers practice intensive dairy farming due to higher population and smaller land sizes (CGONY 2019; CGON 2020). Each county has several milk processors and cooperatives where farmers bulk and market their milk.

### Study design

The study applied a cross-sectional sampling design involving a household survey to understand hygiene practices and laboratory analysis of milk samples for milk microbial quality. The survey was on smallholder dairy farm households within the three counties from July 2019 to December 2019.

### Sampling procedure for the household survey

The study targeted farmers supplying milk to three types of milk processors: government owned, private processor, and farmer or cooperative owned. These processors were also selected based on their willingness to participate in the study. The target population for the household survey was dairy farmers who supplied milk directly to the selected processors in the three counties in 2018. A list of these farmers was obtained from each processor and exhaustive sampling (selecting the population since it was small) was done.

### Sampling of milk for microbial analysis

Raw milk samples were collected from three counties: Bomet, Nyeri, and Nakuru. Milk samples were collected along four major channels:

1. Farmers who supplied milk directly to processors (direct suppliers).
2. Traders who bulked milk from several farmers and transported it to cooperatives.
3. Cooperatives which delivered bulked milk from farmers to processors using their own means of transportation.
4. Cooperatives from which processors collected bulked milk using their own transportation tankers.

However, not all four channels were found in each of the three counties. Bomet had direct suppliers and cooperatives (coop 1) and (coop 2) which both had coolers. Nakuru had direct suppliers, traders, and two cooperatives (coops 3 and 4) which both had coolers. Nyeri had direct suppliers, a cooperative (coop 5) and (coop 6) which did not have coolers and (coop 7) which had a cooler. There were two types of cooperatives: those with coolers (coops 1, 2, 3, 4, and 7) and those without (coops 5 and 6). The separation of cooperatives between those with coolers and those without was to check if the use of coolers had an effect on microbial growth particularly listeria monocytogenes which has been shown to grow in temperatures of 4 °C.

An exhaustive sampling, i.e. selection of the population was done for direct suppliers where 26 samples were collected in Bomet, 18 in Nakuru, and 26 in Nyeri. Exhaustive sampling was also done for traders and cooperatives who supplied milk to the three processors. Three samples were obtained from traders in Nakuru. Three samples were obtained from each cooperative that possessed coolers and two samples were obtained from each cooperative without coolers. A total of 92 samples were obtained: 32 from Bomet, 27 from Nakuru, and 33 from Nyeri as shown in Table 1.

**Table 1:** Sampling distribution for the three counties

County No. of samples	Bomet	Nakuru	Nyeri	Total
Direct suppliers	26	18	26	70
Coop with cooler	6	6	3	15
Coop without cooler	0	0	4	4
Traders	0	3	0	3
Total	32	27	33	92

### Ethical considerations

Local chiefs, who are the relevant and highest government authority in each location and sublocation where the study was carried out, were consulted before beginning the household survey. Interviews and milk samples were obtained only from farmers who consented. Farmers, cooperatives, and transporters were assured of the confidentiality of the data obtained since individual names are not included in the paper.

### Interviews of direct suppliers of milk in the counties

A pretested semi-structured questionnaire was administered to farmers to assess knowledge and practices regarding milk hygiene. In addition, direct observations were carried out at the farms on farm hygiene, dairy herd management, cleanliness of milking equipment, and farm personnel. A total of 73 randomly sampled milk suppliers were interviewed at the household level, comprising 27, 23, and 23 from Bomet, Nakuru, and Nyeri counties, respectively.

### Procedure for obtaining milk samples

**Fig. 2:**
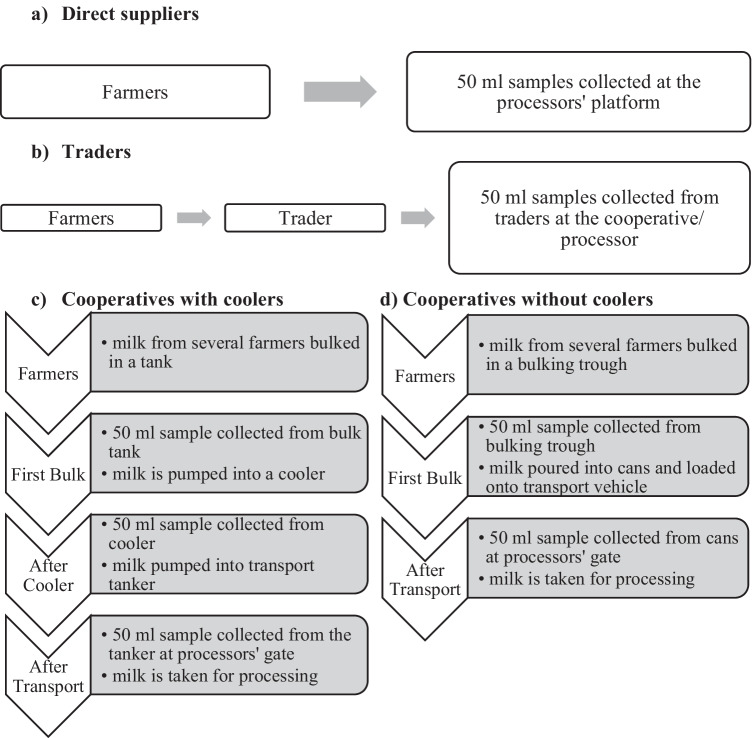
Procedure for obtaining milk samples

All samples were aseptically collected after thorough stirring of the cans, containers, troughs, coolers, and tankers. The samples were collected as shown in Fig. 2.

Milk samples were tightly closed, labelled, and immediately kept in a cool box. They were then transported to the University of Nairobi, Department of Food Science Nutrition and Technology laboratory, where they were stored at 4 °C and analysed within 24 h.

### Microbial analyses

#### Sample preparation

**Fig. 3:**
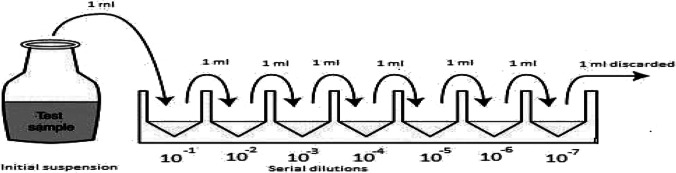
Procedure adopted for serial dilution of samples

Serial dilutions were prepared according to ISO 6887–1 procedure (ISO, 1999). To obtain 15% Buffered Peptone Water (BPW), 15 g of BPW powder was dissolved in 1 l of distilled water according to the manufacturer’s instructions (OXOID® Ltd., Basingstoke, Hampshire, England) and sterilised in the autoclave. Samples were removed from cold storage and allowed for 30 min to attain room temperature. They were then thoroughly shaken and using a sterile pipette, 1 ml of the sample was transferred into a sterile falcon tube containing 9 ml of BPW (10^−1^ dilution), which was followed by serial dilutions as shown in Fig. 3. The procedure was repeated up to 10^−7^ dilution and in the last dilution, 1 ml of inoculum was discarded. The dilutions were mixed thoroughly before they were used to enumerate: TVC, *E. coli*, *S. aureus*, and *L. monocytogenes*.

#### Enumeration of total viable counts

Total viable counts were enumerated as per ISO 4833 (ISO, 2001). Dilutions of 10^−5^ to 10^−7^ of homogenate samples were poured into sterile Petri dishes in duplicate and sterile Standard Plate Count Agar was added. Plates were covered, gentle sufficient shaking was done, and after drying, they were inverted and incubated at 37 °C for 24 h. A colony counter was used to count plates with colonies ranging from 30 to 300, which were expressed as colony-forming units per ml of the sample (CFU/ml).

#### Enumeration of Staphylococcus aureus

*Staphylococcus aureus* was enumerated as per ISO 6888–1:1999 (ISO, 1999). Dilutions of 10^−2^ to 10^−4^ of homogenate samples were pipetted on the surface of previously dried Baird-Parker agar plates in duplicates and spread with a sterile bent glass rod. Plates were incubated at 37 °C for 24 h. Enumeration was done using a colony counter where the colony-forming units were expressed per ml of the sample (CFU/ml). The colonies were identified based on colour which was black and shiny, with narrow white margins, surrounded by clear zones extending into the opaque medium.

#### Enumeration of Escherichia coli

*Escherichia coli* was enumerated as per ISO 16649–2:2001 (ISO, 2001). Dilutions of 10^−2^ to 10^−4^ of homogenate samples were pipetted on to sterile plates in duplicates, sterile HiCrome agar was added, and gentle sufficient shaking was done. After drying, the plates were inverted and incubated at 37 °C for 24 h. Enumeration was then done using a colony counter where colony-forming units were expressed per ml of sample (CFU/ml) for colonies which had bluish green colour.

#### Enumeration of Listeria monocytogens

*Listeria monocytogens* was enumerated as per ISO 10560 (ISO, 2001). Dilutions of 10^−2^ to 10^−4^ of homogenate samples were pipetted onto the surface of dried *Listeria* chromogenic agar in duplicate and spread with a sterile bent glass rod. Plates were inverted and incubated at 37 °C for 24 h. Enumeration was done using a colony counter for colony-forming units on colonies which had blue to blue-green colour and expressed per ml of sample (CFU/ml).

### Data analysis

Household survey data was analysed using descriptive statistics where Statistical Package for the Social Sciences (SPSS) version 22 (SPSS Inc., Chicago, IL, USA) was used to analyse farmer data on hygienic practices. Significant differences in socio-demographic characteristics, knowledge, and hygiene practices in the various counties were tested using one-way ANOVA at 95% confidence interval. Associations between socio-demographic characteristics and knowledge on hygiene practices in the various counties were tested using chi^2^. Laboratory data was analysed using GenStat version 15 where mean differences were separated by the least significant difference procedure using Tukey’s formula.

## Results

### Socio-demographic characteristics of the respondents

Socio-demographic characteristics of direct suppliers are as represented in Table 2. There was no significant difference (*p* > 0.05) in gender, age of respondents in all categories, marital status in terms of being married or living with spouse, and level of education of respondents at the elementary and middle school level. However, there was a significant difference (*p* < 0.05) in the farming system practised in the counties where most farmers (92.3%) in Bomet practiced semi-intensive farming while 83.3% and 100% of farmers in Nakuru and Nyeri respectively practised intensive farming.

**Table 2:** Socio-demographic characteristics (percent respondents) of direct suppliers in Bomet, Nakuru, and Nyeri counties

Characteristic	Bomet	Nakuru	Nyeri	Total
*Gender*				
Male	61.5	44.4	65.4	57.5
Female	38.5	55.6	34.6	42.5
*Age (years)*				
18–35	26.9	27.8	11.5	22.1
35–50	42.3	38.9	38.5	39.9
> 50	34.6	33.3	50.0	38.0
*Education level*				
No formal education	3.8	22.2	0.0	8.2
Elementary	30.8	38.9	50.0	39.7
Middle school	50.0	38.9	19.2	37.0
High school	11.5	0.0	30.8	15.1
University	0.0	0.0	0.0	0.0
*Marital status*				
Married	80.8	77.8	73.1	76.7
Single	19.2	0.0	11.5	12.3
Divorced	0.0	0.0	3.8	1.4
Widow/er	0.0	22.2	11.5	9.6
*Farm system*				
Intensive	7.7	83.3	100	60.3
Semi-intensive	92.3	16.7	0.0	39.7

### Milk handling and hygienic practices

**Fig. 4:**
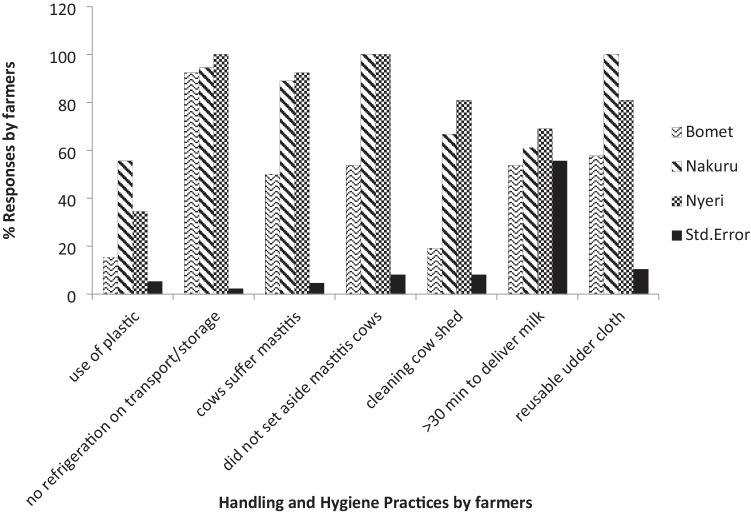
Handling and hygiene practices by farmers in Bomet, Nakuru, and Nyeri counties

Different handling and hygiene practices were observed in farmers in the three counties (Fig. 4). There was a significant difference (*p* < 0.05) in the type of milking containers used by farmers in the three counties. Most used plastic containers for milking and transportation of milk with Nakuru recording the highest where 56% of the farmers used plastic. There was also a significant difference (*p* < 0.05) in cleaning of the sheds, use of reusable cleaning cloths to clean udders, and setting aside cows with mastitis in the three counties. While the farmers in Nyeri and Nakuru applied more hygienic practices in cleaning the cow shed and udders in comparison to farmers in Bomet, about half of the farmers in Bomet set aside mastitis cows, while none of their counterparts in Nyeri and Nakuru did the same. These practices are important in that they directly affect milk quality. There was no significant difference (*p* > 0.05) in refrigeration of milk by farmers and the time they took to deliver milk to the processors.

In Bomet, there was a significant association between the level of education of the respondents and the type of milking can they used. Additionally, in Bomet, there was a significant association between the type of farming practised by the respondents and cleaning of sheds.

Cleaning practices for milk containers and udder were similar in Nakuru and Nyeri where more than 80% of the farmers always cleaned their containers and used a cleaning cloth for the udder (Table 3). This was different in Bomet which brought about a significant difference (*p* > 0.05) in the cleaning of milk containers in the three counties. It was found that all the farmers in Nyeri always cleaned milking containers; however, most of them (41.4%) used water only to clean the containers while 20.7% used water with disinfectants. Cleaning of udders varied significantly (*p* < 0.05) in the three counties where almost half (43.5%) of farmers in Bomet used their bare hands to clean udders while most farmers in Nakuru and Nyeri used a cleaning cloth.

**Table 3:** Cleaning practices of farmers (percent respondents) in the various counties

County Hygienic practice	Bomet	Nakuru	Nyeri	Total
*Source of water*				
Tap/pipe	10.7	21.4	80.2	35.6
Well	50.2	46.5	11.3	35.6
River	39.1	32.1	8.5	26.0
*Frequency of cleaning milking containers*				
Always	80.7	100	100	93.2
Most often	19.2	0.0	0.0	6.8
*Cleaning udder*				
Hand	42.3	0.0	9.2	17.8
Cleaning cloth	57.7	100	80.8	82.2

In Nyeri, there was a significant association between the gender of respondents and cleaning of milking containers.

### Knowledge on hygiene and milk handling practices

Table 4 shows that 38.4% of all interviewed farmers found it ok to feed spoiled feed to their cows. This practice was also found by Kiama et al. (2016) in Kenya, where farmers commonly fed spoiled maize and food to their animals thereby increasing the risk of exposure of mycotoxins to the animals. There was no significant difference (*p* > 0.05) on the dangers of feeding spoilt feed to cows in the counties. Notably, more than half (58.6%) of farmers in Nyeri said that it was okay to feed spoiled feed to cows which would be an issue of concern on milk quality (Table 4). There was no significant difference (*p* > 0.05) in hygienic milking and delivering milk promptly as ways of ensuring milk did not spoil in the three counties. There was also no significant difference (*p* > 0.05) in knowledge of mastitis where most farmers in the counties knew the disease and could detect it in cows. There was a significant difference (*p* < 0.05) in adulteration and density as causes of milk rejection in the three counties. Most farmers (34.6%) in Bomet thought that milk adulteration would lead to rejection on delivery while 44.5% and 41.3% in Nakuru and Nyeri, respectively, thought that addition of water to alter the density would lead to milk rejection on delivery.

**Table 4:** Knowledge on hygiene and handling practices by farmers (percent respondents) in the various counties

County Knowledge on hygiene	Bomet	Nakuru	Nyeri	Total
*Is it okay to feed spoilt feed to cows*				
Yes	30.4	32.0	58.6	38.4
No	69.6	68.0	41.4	61.6
*How do you ensure that milk does not get spoiled during storage*				
Boiling	15.4	14.6	19.2	15.1
Cover container	7.6	19.7	42.3	23.3
Deliver promptly	34.6	5.9	15.4	19.2
Hygienic milking	3.8	17.6	3.8	8.2
Store in a cool place	15.4	5.9	3.8	8.2
Do nothing	23.1	36.3	15.4	26.0
*What are the causes of milk rejection on delivery*				
Acidity	3.8	5.6	3.8	4.1
Organoleptic (smell, temperature, visible foreign particles)	17.3	5.6	3.8	9.6
Low density (water addition)	15.4	44.5	41.3	30.1
Adulteration (using other substances except water)	34.6	12.5	23.2	23.3
Others	11.5	11.5	12.5	17.8
Don’t know	17.3	9.2	15.4	15.1
*Can you detect mastitis in cows*				
Yes	95.7	84.0	86.2	89.0
No	4.3	16.0	13.8	11.0

In Nakuru, there was an association between the level of education of respondents and their knowledge on the causes of milk rejection on delivery.

### Microbial quality of milk in different collection channels

#### Microbial quality of milk from Bomet County

In the cooperative channel, after cooler milk samples from coop 2 recorded the highest TVC (8.1 log cfu/ml), while after cooler samples from coop 1 had the lowest counts of 6.8 log cfu/ml (Table 5). There was, however, no significant difference (*p* > 0.05) among the samples along the channel. The level of TVC in all counties exceeded the 6.3 log cfu/ml set standard (EAC 2018).

**Table 5:** Microbial quality of milk in Bomet County along the collection channels in log cfu/ml

Microbial attribute Collection channels	*S. aureus*	*E. coli*	*L. monocytogenes*	TVC
Direct suppliers	5.315 ± 0.6^b^	3.268 ± 1.2^b^	5.641 ± 0.8^a^	7.612 ± 0.6^a^
Coop 1 first bulk	3.518 ± 0.0^a^	2.739 ± 0.1^ab^	6.622 ± 0.0^a^	6.851 ± 0.0^a^
Coop 1 after cooler	3.643 ± 0.1^a^	0 ± 0^a^	6.874 ± 0.0^a^	6.771 ± 0.0^a^
Coop 2 first bulk	5.058 ± 0.0^ab^	3.498 ± 0.0^b^	6.005 ± 0.0^a^	7.924 ± 0.0^a^
Coop 2 after cooler	4.475 ± 0.1^ab^	5.071 ± 0.0^b^	6.249 ± 0.0^a^	8.095 ± 0.0^a^
Coop 2 after transport	4.198 ± 0.0^ab^	4.541 ± 0.1^b^	6.848 ± 0.0^a^	7.686 ± 0.0^a^

Milk samples from direct suppliers had the highest *S. aureus* counts (5.3 log cfu/ml) while first bulk milk samples from coop 1 had the lowest counts (3.5 log cfu/ml). There was a significant difference (*p* < 0.05) in the counts in milk samples supplied directly by farmers and those from coop 1. However, there was no significant difference (*p* > 0.05) in milk samples supplied directly by farmers and those from coop 2. Apart from first bulk and after cooler milk samples of coop 1 which met the set standard of 4.7 log cfu/ml (EAC 2018), the rest exceeded the standards.

Results are mean of duplicate samples ± standard deviation; *TVC*, total viable counts

Means with the same letters in superscript in the same column are not significantly different at *p*<0.05

KEBS standards: *S. aureus* (4.7 log cfu/ml), *E. coli* (4.0 log cfu/ml), *L. monocytogenes* (2.0 log cfu/ml), TVC (6.3 log cfu/ml).

*E. coli* counts varied significantly depending on the collection channel with milk from after cooler samples of coop 2 recording the highest counts (5.1 log cfu/ml) while after cooler samples from coop 1 had the lowest counts (0 log cfu/ml). There was no significant difference (*p* > 0.05) between first bulk and after cooler milk samples from coop 1 (*p* < 0.05). All samples along the channel met the set standards of 4 log cfu/ml (EAC 2018) with the exception of after cooler samples of coop 2.

There were no significant (*p* > 0.05) variations in *L. monocytogenes* counts along the collection channels. Milk samples from direct suppliers had the lowest counts (5.6 log cfu/ml), while after cooler samples from coop 1 had the highest counts (6.9 log cfu/ml). It was noted that from the two cooperatives, after cooler and after transport milk samples had higher counts than first bulk milk samples.

#### Microbial quality of milk from Nakuru County

Total viable counts varied significantly (*p* < 0.05) where after transport samples from coop 4 had the highest counts (9.5 log cfu/ml) while after transport samples from coop 3 had the lowest counts of 7.4 log cfu/ml (Table 6). All samples exceeded the set standards of 6.3 log cfu/ml (EAC 2018).

**Table 6:** Microbial quality of milk in Nakuru in the along the collection channels in log cfu/ml

Microbial attribute Collection channel	*E. coli*	*L. monocytogenes*	*S. aureus*	TVC
Direct suppliers	3.948 ± 1.2^ab^	5.789 ± 0.5^b^	4.734 ± 1.1^a^	8.378 ± 1.0^abc^
Traders	4.469 ± 0.8^abc^	4.605 ± 2.5^b^	5.11 ± 1.2a	9.13 ± 0.3^bd^
Coop 3 first bulk	6.01 ± 0.0^abc^	7.039 ± 0.0^b^	6.258 ± 0.0^a^	9.444 ± 0.0^abcd^
Coop 3 after cooler	6.348 ± 0.0^ac^	6.585 ± 0.0^b^	6.276 ± 0.0^a^	9.193 ± 0.0^abcd^
Coop 3 after transport	3.379 ± 0.1^a^	0 ± 0.0^a^	3.726 ± 0.1^a^	7.391 ± 0.0^a^
Coop 4 first bulk	4.911 ± 0.0^abc^	5.885 ± 0.1^b^	6.05 ± 0.0^a^	8.325 ± 0.0^ab^
Coop 4 after cooler	5.94 ± 0.1^abc^	6.017 ± 0.0^b^	6.082 ± 0.0^a^	8.927 ± 0.0^abcd^
Coop 4 after transport	5.949 ± 0.0^abc^	6.626 ± 0.1^b^	6.299 ± 0.0^a^	9.458 ± 0.0^abcd^

Results are mean of duplicate samples ± standard deviation; *TVC*, total viable counts

Means with common letters in superscript in the same column are not significantly different at *p*<0.05

KEBS standards: *S. aureus* (4.7 log cfu/ml), *E. coli* (4.0 log cfu/ml), *L. monocytogenes* (2.0 log cfu/ml), TVC (6.3 log cfu/ml)

There were no significant (*p* > 0.05) variations in *S. aureus* counts along the collection channels. After transport milk samples from coop 4 had the highest counts (6.3 log cfu/ml) together with after cooler and first bulk samples from coop 3 (6.3 log cfu/ml). After transport samples from coop 3 had the lowest counts (3.7 log cfu/ml) and the only one that met the set standard of 4.7 log cfu/ml.

There were no significant (*p* > 0.05) variations in *E. coli* counts along the collection channels. After cooler milk samples from coop 3 had the highest counts (6.3 log cfu/ml) while after transport samples from the same cooperative had the lowest counts (3.4 log cfu/ml). Milk samples from direct suppliers, traders, and coop 3 after transport are the only ones that met the set standards of 4 log cfu/ml.

There was a significant (*p* < 0.05) variation in after transport samples from coop 3 with the rest of the samples in *L. monocytogenes* counts along the collection channels. First bulk milk samples from coop 3 had the highest counts (7.0 log cfu/ml), while after transport samples from the same cooperative had the lowest counts (0 log cfu/ml).

#### Microbial quality of milk in Nyeri County

There were no significant (*p* > 0.05) variations in TVC along the collection channels (Table 7). First bulk milk samples from coop 5 had the highest counts (9.4 log cfu/ml) while first bulk samples from coop 7 had the lowest counts (8.3 log cfu/ml). All samples exceeded the set standard of 6.3 log cfu/ml (EAC 2018).

**Table 7:** Microbial quality of milk in Nyeri along the collection channels in log cfu/ml

Microbial attribute Collection centre	*E. coli*	*L. monocytogenes*	*S. aureus*	TVC
Direct suppliers	5.068 ± 1.7^a^	5.552 ± 1.3^a^	4.463 ± 0.7^a^	8.537 ± 0.6^a^
Coop 5 first bulk	6.291 ± 0.0^a^	7.135 ± 0.0^a^	6.052 ± 0.0^b^	9.438 ± 0.0^a^
Coop 5 after transport	7.172 ± 0.0^a^	5.148 ± 0.1^a^	6.035 ± 0.0^b^	9.365 ± 0.0^a^
Coop 6 first bulk	4.017 ± 0.1^a^	5.611 ± 0.1^a^	6.394 ± 0.0^b^	9.304 ± 0.0^a^
Coop 6 after transport	3.952 ± 0.1^a^	5.724 ± 0.0^a^	5.208 ± 0.0^ab^	9.32 ± 0.0^a^
Coop 7 first bulk	3.239 ± 0.3^a^	5.707 ± 0.0^a^	4.536 ± 0.1^ab^	8.284 ± 0.1^a^
Coop 7 after cooler	3 ± 0^a^	8.017 ± 0.0^a^	4.573 ± 0.0^ab^	8.442 ± 0.0^a^
Coop 7 after transport	4.677 ± 0.1^a^	7.851 ± 0.0^a^	4.806 ± 0.0^ab^	9.037 ± 0.0^a^

Results are mean of duplicate samples ± standard deviation; *TVC*, total viable counts

Means with common letters in superscript in the same column are not significantly different at *p*<0.05

KEBS standards: *S. aureus* (4.7 log cfu/ml), *E. coli* (4.0 log cfu/ml), *L. monocytogenes* (2.0 log cfu/ml), TVC (6.3 log cfu/ml)

Milk samples from direct suppliers varied significantly (*p* < 0.05) with samples from coop 5. First bulk samples from coop 6 had the highest *S. aureus* counts (6.4 log cfu/ml), while samples from direct suppliers had the lowest counts (4.5 log cfu/ml). Milk samples from direct suppliers, coop 7 first bulk, and after cooler are the only ones that had counts below the set standard of 4.7 log cfu/ml (EAC 2018) while the rest exceeded the set standards.

There were no significant (*p* > 0.05) variations in *E. coli* counts along the collection channels. After transport samples from coop 5 had the highest counts (7.2 log cfu/ml), while after cooler samples from coop 7 had the lowest counts (3 log cfu/ml). Milk samples from direct suppliers, coop 5 first bulk, and coop 5 after transport exceeded the set standard of 4 log cfu/ml (EAC 2018) while the rest met the set standards.

There were no significant (*p* > 0.05) variations in *L. monocytogenes* counts along the collection channels. After cooler milk samples from coop 7 had the highest counts (8.0 log cfu/ml) followed by after transport samples from the same cooperative (7.9 log cfu/ml), while after transport samples from coop 5 had the lowest counts (5.1 log cfu/ml).

Cooperatives 5 and 6 had no coolers and they recorded higher TVC, *S. aureus*, and *E. coli* counts than coop 7 which had a cooler. On the other hand, coop 7 had higher *L. monocytogenes* counts than coops 5 and 6.

### Comparison of milk quality across the studied counties

Nakuru County recorded the highest mean TVC of 8.7 log_10_ cfu/ml, Nyeri had the highest *E. coli* mean counts of 4.97 log_10_ cfu/ml, and Bomet recorded the highest mean counts of 5.13 and 5.78 log_10_ cfu/ml for *S. aureus* and *L. monocytogenes* respectively (Table 8).

**Table 8:** General microbial quality of milk in different counties in log cfu/ml

**Microbial attribute** **Collection channel**	**TVC**	***E. coli***	***L. monocytogenes***	***S. aureus***
Bomet	7.588 ± 0.6^a^	3.253 ± 1.3^a^	5.783 ± 0.8^a^	5.132 ± 0.7^b^
Nyeri	8.641 ± 0.6^b^	4.973 ± 1.7^b^	5.744 ± 1.3^a^	4.656 ± 0.8^a^
Nakuru	8.72 ± 0.8^b^	4.449 ± 1.2^b^	5.298 ± 1.9^a^	5.092 ± 1.2^b^

Results are mean of duplicate samples ± standard deviation; *TVC*, total viable counts

Means with common letters in superscript in the same column are not significantly different at *p*<0.05

KEBS standards: *S. aureus* (4.7 log cfu/ml), *E. coli* (4.0 log cfu/ml), *L. monocytogenes* (2.0 log cfu/ml), TVC (6.3 log cfu/ml)

## Discussion and conclusion

### Handling and hygiene practices

In Bomet, there was a significant association between the level of education of respondents and the type of milking container they used (Table 3). Most respondents who had no formal education and those who went up to elementary school mainly used plastic containers while those who attained middle and high school education mainly used aluminium. Similar results were observed in Ethiopia by Kebede and Megerrsa (2017) where more educated farmers had better farm hygiene practices. However, this was different in Nyeri and Nakuru where the level of education of the respondents did not influence the type of container they used to store and transport milk.

Additionally, in Bomet, there was a significant association between the farming system practised by respondents and cleaning of sheds. Farmers who practised intensive farming cleaned sheds more often compared to those who practised semi-intensive farming which was not the case in Nakuru and Nyeri counties. Similar results were observed in Nakuru and Laikipia counties in Kenya by Nyokabi et al. (2021) where more than 90% of farmers practiced extensive farming and hardly cleaned the sheds.

In Nyeri County, it was noted that female respondents took better care of their milking containers as they were more likely to use both soap and water in cleaning milking containers while male respondents mostly used water only. This tendency was however not observed in Bomet and Nakuru counties. In Nakuru, there was significant association between the level of education of respondents and their knowledge on the causes of milk rejection on delivery. Most respondents who had no formal education did not know the causes of rejection, while those who had attained elementary, middle, and high school education cited various causes for rejection which was not the case in Bomet and Nyeri counties. These results are different from those found by Ndungu et al. (2016a) who observed that farmers were aware about the causes of milk rejection from their cooperatives.

### Total viable counts

TVC results in this study exceeded the set standards of 6.3 log_10_ cfu/ml or 2 million cfu/ml (EAC 2018). This could be due to various unhygienic milking and handling practices at the farm. High TVC value is an indication of raw milk that is not suitable for consumption which also indicates an increased risk of the presence of pathogenic microorganisms (Knight-jones et al. 2016). These food-borne pathogens can persist in biofilms resulting in contamination of processed milk products, especially in cases where inadequate pasteurization is done (Rola et al. 2016). A common observation in the three counties was that farmers held milk at the farm after milking without refrigeration to attend to other chores. The longer holding time in warm tropical weather results in rapid multiplication of bacteria, hence high microbial counts on delivery (Alonso et al. 2018; Lindahl et al. 2018). Nakuru County had the highest mean TVC which could have resulted from the rampant use of plastic containers for milking and storage of milk. More than half (55.6%) of farmers in Nakuru used plastic containers compared to 13.4% and 34.6% of farmers in Bomet and Nyeri respectively. Plastic containers adhere to milk residues making them difficult to clean compared to aluminium containers. This shows an improvement from the situation recorded in a previous study (Ndungu et al. 2016b) where 90% and 49% of farmers in Nakuru and Nyandarua counties respectively were found to be using plastic containers for transportation of milk. The improvement could have resulted from various trainings which farmers from Nakuru County received in the last years (Ndambi et al. 2019). Ndungu et al. (2016b) further observed high mean TVC: 6.455, 6.276, 6.369, and 7.138 log_10_ cfu/ml from milk collected from individual cans, collection routes, milk cooler, and tanker respectively in Nakuru County. This study also noted that all (100%) farmers in Nakuru often used a reusable cleaning cloth to wipe hands, equipment, and udders of different cows compared to 57.7% and 80.8% of farmers in Bomet and Nyeri respectively (Table 3). This is a poor handling practice due to transfer of bacteria from hand to udder, hand to equipment, or between udders of various cows resulting in contamination of milk. Most farmers indicated that they rarely changed these cloths which could be sources of microbial contamination especially when not well cleaned as observed in another study in Nairobi by Wanjala et al. (2018).

High microbial counts were observed in the first bulk and after cooler milk samples from coop 3, although milk from the same cooperative had the lowest microbial counts after it was transported to the processor in a chilling tank. This raises concerns and could be due to a number of reasons including the addition of hydrogen peroxide which has microcidal properties, thus lowering the number of microorganisms in the milk that arrived at the processor (Wallace 2015). Micro-organisms in milk of high bacteria load could form toxins which are heat resistant and can survive through processing making them present in the end product (Ozer and Yaman 2014; Meunier-Goddik and Sandra 2011).

Cooperatives without coolers had higher microbial counts than those with coolers. Long holding time at these cooperatives with no cooling could encourage rapid microbial growth (Velázquez-ordoñez et al. 2019). The increase in microbial growth observed between the first bulk and after cooler samples for cooperatives with coolers could be due to poor cooling efficiency, since it took over 3 h to cool milk from around 20 to 4 °C. Instant coolers which rapidly cool milk compared to conventional coolers thus reducing the multiplication of bacteria are recommended for cooperatives (Ndungu et al. 2016b). In addition, quality-based milk payment systems could be promoted as they would stimulate farmers to improve hygienic practices (Özkan Gülzari et al. 2020; Ndambi et al. 2019).

### Staphylococcus aureus

Most of the *Staphylococcus aureus* counts in this study exceeded the set standards where the acceptable limit is 10,000 cfu/ml or 4 log cfu/ml (EAC 2018). It was observed in the three counties that over 95% of farmers milk manually or by hand, which could be a source of *S. aureus* contamination especially when hands are not properly cleaned considering that humans are carriers of the microorganism (Orregård 2013). Direct suppliers in Bomet recorded the highest *S. aureus* counts which could be as a result of hand cleaning of the udders as done by 42.3% of farmers in the county compared to 0% and 9.2% of farmers in Bomet and Nyeri respectively (Table 3). It was also observed that these farmers washed their hands simply with cold water before milking which does not guarantee effective cleaning of hands. This agrees with a study done by Orregård (2013) in Kiambu County in Kenya where high *S. aureus* counts in 70% of the samples were attributed to hand cleaning of the udders. *S. aureus* is an organism associated with mastitis (Wallace 2015) which explains the high counts in Nakuru and Nyeri counties where all (100%) farmers did not set aside cows with mastitis resulting in contamination of milk compared to 53.8% of farmers in Bomet.

Cooperatives in Nakuru County recorded the highest *S. aureus* counts. Untidy platforms, inefficient cleaning of the coolers, and poor personnel hygiene as observed at the cooperatives could be sources of milk contamination (Enquebaher et al. 2015). Cooperative 5 in Nyeri also recorded high *S. aureus* counts. This cooperative received milk from farmers and held it without cooling for a few hours before it was delivered to the processors still without refrigeration, a practice which results in rapid multiplication of bacteria (Migose et al. 2018). This study agrees with one done by Wanjala et al. (2018) where mean *S. aureus* counts were 5.83, 6.32, and 5.82 log_10_ cfu/ml in raw milk collected from Kenyan rural, urban, and slum areas respectively all exceeding the set standards. However, the results in this study were higher than those found in a study done in Bangladesh where *S. aureus* counts in raw milk samples from farms, chilling centres, and traders were 2.90, 2.77, and 2.78 log cfu/ml respectively (Islam et al. 2016).

### Escherichia coli

High *E. coli* counts in raw milk can be attributed to poor farm or herd hygiene (Gemechu et al. 2015). Direct suppliers in Bomet had high *E. coli* counts which could be attributed to the fact that 80.8% of farmers in the area rarely cleaned sheds or disposed dung compared to 19.2% and 33.3% of farmers in Nyeri and Nakuru respectively, resulting in mud and faeces being sources of contamination within addition, hand cleaning of the udders as practised by 42.3% of farmers in Bomet compared to none (0%) and 9.2% of farmers in Nakuru and Nyeri respectively. This practice does not guarantee efficient cleaning, thus compromising milk quality. High *E. coli* counts in Bomet and Nakuru counties could have resulted from contaminated water. It was observed that 50.2% and 46.5% of farmers in Bomet and Nakuru counties respectively sourced water from wells while 39.1% and 32.1% of farmers from the same counties sourced water from rivers and used the water for cleaning and feeding the cattle without any form of treatment. Farmers in the three counties cited that density or addition of water was a cause of milk rejection on delivery to the processor. Presence of *E. coli* in raw milk samples that were aseptically collected from the three counties can indicate the use of contaminated water in cases of milk adulteration as observed by Amenu et al. (2016) in Southern Ethiopia. The high *E. coli* counts coincide with a study done by Alonso et al. (2018), where the median coliform count of raw milk samples consumed in households in Nairobi was 3 million cfu/ml exceeding the set standard of 50,000 cfu/ml (EAC 2018). In Asia, Koirala (2018) did a study on raw milk samples in Pokhara where total coliform counts of samples at farm level ranged from 0 to 1.2*10^5^ cfu/ml while those from milk collection centres had a mean count of 3.4*10^4^ cfu/ml. Presence of *E. coli* indicates the presence of other coliforms and is an indicator of faecal contamination and thus posing great safety and public health concerns (Wallace 2015). Raw milk which has high *E. coli* contamination develops off-flavour fast even after processing, hence reduced shelf life of dairy products (Reta and Addis 2015).

### Listeria monocytogenes

It has been noted that *L. monocytogenes* is the only *Listeria* that has pathogenic effects in healthy humans. When products such as cheese are made fromraw milk that has been infected with *L. monocytogenes*, bacterial growth occurs resulting in a highly contaminated product which causes listeriosis on consumption (Wallace 2015). Listeriae are commonly found in the environment (Ulusoy and Chirkena 2019). Generally, Bomet County recorded the highest *L. monocytogenes* counts likely because more of their animals were allowed to graze which could be a source of contamination compared to those in Nakuru and Nyeri. The most common source of *L. monocytogenes* infection in dairy cows is from poorly preserved silage (Seyoum et al. 2015). Direct suppliers in Nyeri and Nakuru counties recorded high *L. monocytogenes* counts where 58.6% and 32% of farmers in the respective counties cited that it was okay to give spoiled feed to dairy cows, a practice which results in contamination of milk. Cooperatives in the three counties especially those with coolers recorded high *L. monocytogenes* count for the after cooler and after transport samples. *L. monocytogenes* has the ability to survive in temperatures as low as 4 °C in already contaminated milk; hence, the high numbers of the sample contamination levels in this study were higher compared to those found in Ethiopia where 18.9% of raw milk samples were found to be contaminated by *L. monocytogenes* at the farm level (Seyoum et al. 2015). This could be due to the fact that farmers in Ethiopia mostly practised intensive dairy farming thus minimizing contamination of milk.

Based on the findings of this study, farmers in the three counties did not employ good hygienic practices in dairy management. Milking was done with little consideration on measures to ensure quality. Farmers used plastic containers for milking and storage of milk; they rarely cleaned sheds and did not set aside cows with mastitis. These unhygienic practices resulted in the initial contamination of milk. Handling during transport and at the cooperatives further increased the contamination of milk before it reached the processor. In most samples, the average TVC, *E. coli*, *S. aureus*, and *L. monocytogenes* counts in all counties exceeded the set Kenyan standards. High bacteria counts poses a health risk to consumers considering that most of these microorganisms form toxins which are heat resistant and can survive processing temperatures and conditions making their way to the end product. Constant training for farmers and cooperative personnel on hygienic practices and milk handling is required coupled with improvement of road infrastructure, installation of instant coolers at cooperatives to further reduce the multiplication of bacteria. Moreover, the use of a quality-based system for milk payment could provide farmers incentives to improve milk quality.

## Data Availability

Raw survey data is available upon request.
